# Zinc Levels Modulate Lifespan through Multiple Longevity Pathways in *Caenorhabditis elegans*

**DOI:** 10.1371/journal.pone.0153513

**Published:** 2016-04-14

**Authors:** Jitendra Kumar, Tracy Barhydt, Anjali Awasthi, Gordon J. Lithgow, David W. Killilea, Pankaj Kapahi

**Affiliations:** 1 The Buck Institute for Research on Aging, Novato, California, United States of America; 2 Department of Biological Sciences, Birla Institute of Technology and Science, Rajasthan, India; 3 Nutrition & Metabolism Center, Children’s Hospital of Oakland Research Institute, Oakland, California, United States of America; 4 DBT-PU-IPLS Programme, Department of Botany/Biotechnology, Patna University, Patna- 800005, Bihar, India; CSIR-Central Drug Research Institute, INDIA

## Abstract

Zinc is an essential trace metal that has integral roles in numerous biological processes, including enzymatic function, protein structure, and cell signaling pathways. Both excess and deficiency of zinc can lead to detrimental effects on development and metabolism, resulting in abnormalities and disease. We altered the zinc balance within *Caenorhabditis elegans* to examine how changes in zinc burden affect longevity and healthspan in an invertebrate animal model. We found that increasing zinc levels *in vivo* with excess dietary zinc supplementation decreased the mean and maximum lifespan, whereas reducing zinc levels *in vivo* with a zinc-selective chelator increased the mean and maximum lifespan in *C*. *elegans*. We determined that the lifespan shortening effects of excess zinc required expression of DAF-16, HSF-1 and SKN-1 proteins, whereas the lifespan lengthening effects of the reduced zinc may be partially dependent upon this set of proteins. Furthermore, reducing zinc levels led to greater nuclear localization of DAF-16 and enhanced dauer formation compared to controls, suggesting that the lifespan effects of zinc are mediated in part by the insulin/IGF-1 pathway. Additionally, zinc status correlated with several markers of healthspan in worms, including proteostasis, locomotion and thermotolerance, with reduced zinc levels always associated with improvements in function. Taken together, these data support a role for zinc in regulating both development and lifespan in *C*. *elegans*, and that suggest that regulation of zinc homeostasis in the worm may be an example of antagonistic pleiotropy.

## Introduction

Zinc is an essential micronutrient involved in the structure, regulation, and activity for thousands of proteins, and participates in many biological processes such as redox regulation and signal transduction [1–6]. Zinc-dependent functions are widespread within the multicellular organism but especially in the central nervous system, immune system, skeletal and reproductive system [7–13]. Consequently, severe zinc deficiency in humans leads to functional defects in growth and development, hypogonadism, dermatitis, delayed wound healing, and decreased immune function [7–9,11–19]. Conversely, exposure to elevated zinc can also have toxic effects [20,21], which may be in part due to the competitive displacement of other trace metals from their binding sites or adventitious binding to protein regions not normally involved with metal, both leading to protein dysfunction [20–24]. Excess zinc can also promote non-physiological protein aggregation. Hence, maintaining optimal zinc balance is vital for proper physiological functions. Animals prevent zinc deficiency or toxicity through a range of regulatory mechanisms, including altering expression of zinc transporters in response to changing levels of zinc [25–27], chelation of zinc by small molecules such as glutathione or proteins such as metallothionein [21,23,25–28], and sequestration of zinc within intracellular organelles [29–31]. Multiple studies have shown that zinc homeostasis can be efficiently regulated by these mechanisms, even in the face of wide ranges of dietary or environmental zinc exposure [23–31].

Like all animals, the free-living nematode *Caenorhabditis elegans* requires zinc for survival, and the homeostasis of zinc has been well studied in *C*. *elegans* [32–35]. Zinc metabolism in the worm has been shown to be regulated by the highly conserved cation diffusion facilitators (CDF) transporter, Zrt and Irt-like proteins (ZIP) transporter, and metallothionein (MT) protein families [32,33,36–40]. CDF-1, CDF-2 and SUR-7 are the known members of CDF family transporters [32,36–38]. These transporters are thought to sequester or move zinc throughout the body. The expression of zinc transporters is increased at high level of zinc exposure, suggesting they can also move zinc back into the gut and out of the animal [39]. Worms with loss of function mutations of these zinc transporters show growth defects and abnormal zinc content compared to wildtype animals [34,39]. Furthermore, CDF transporter mutants display heightened toxicity towards increasing concentration of zinc [39]. The CDF-1 transporter is similar to vertebrate ZnT-1, with the highest localization in intestinal cells [36–38]. The CDF-2 transporter is similar to vertebrate ZnT-2, which is more abundant in vesicles [36,38], suggesting an important role in zinc storage. The SUR-7 transporter, predominantly expressed in the endoplasmic reticulum, may function to sequester zinc ions in cellular organelle [38]. Further analysis of CDF-1 and CDF-2 suggests that these transporters have antagonistic functions in mediating zinc content *in vivo* [38]. The MT protein family comprises several small molecular weight, thiol-rich proteins shown to sequester zinc and other metals in vivo. Deletion of MT proteins result in increased Zn accumulation and increased sensitivity to high zinc levels [33]. Despite a detailed knowledge of zinc regulatory proteins, only a few studies have examined the effects of imbalances in zinc levels upon the development, metabolism, and aging of worms [32–35].

In this paper, we further characterized the effect of zinc status on *C*. *elegans* lifespan and healthspan. *C*. *elegans* have well-established culture conditions that permit manipulation of dietary zinc [34,36,39,41]. We found zinc supplementation cause a decrease in lifespan, which required DAF-16, HSF-1, SKN-1. In contrast, reductions in zinc levels resulted in an increased lifespan, which was in part dependent on DAF-16, HSF-1, SKN-1. Furthermore, we also examined the effect of alteration in zinc burden on key processes in development and aging, such as dauer formation and protein aggregation. Zinc balance appears to be critical for worm development, and it may limit lifespan through antagonistic pleiotropic mechanisms involving multiple longevity pathways.

## Results

### Zinc availability alters *C*. *elegans* lifespan

To characterize the effects of zinc on lifespan, wildtype *C*. *elegans* populations were cultured on noble agar minimal media (NAMM) containing ZnSO_4_ added to the *E*. *coli* OP50 bacteria. We first tested for toxicity of the supplemental zinc by monitoring growth and body size development of the worms. Worms cultured with zinc supplemented up to 500μM demonstrated similar growth and body size compared to wildtype populations (S1 Fig). Compared to wildtype populations (1.03±0.16 mm), worms treated with 200μM zinc were on average 0.93±0.23 mm, 500μM zinc were 0.99±0.27 mm, and 1mM zinc were 0.82±0.17 mm long. Higher concentrations of up to 5mM zinc showed significant reductions in growth and body size, and obvious increases in population death (data not shown). Therefore, we used 500μM as the maximum zinc dose for all future experiments.

**Fig 1:**
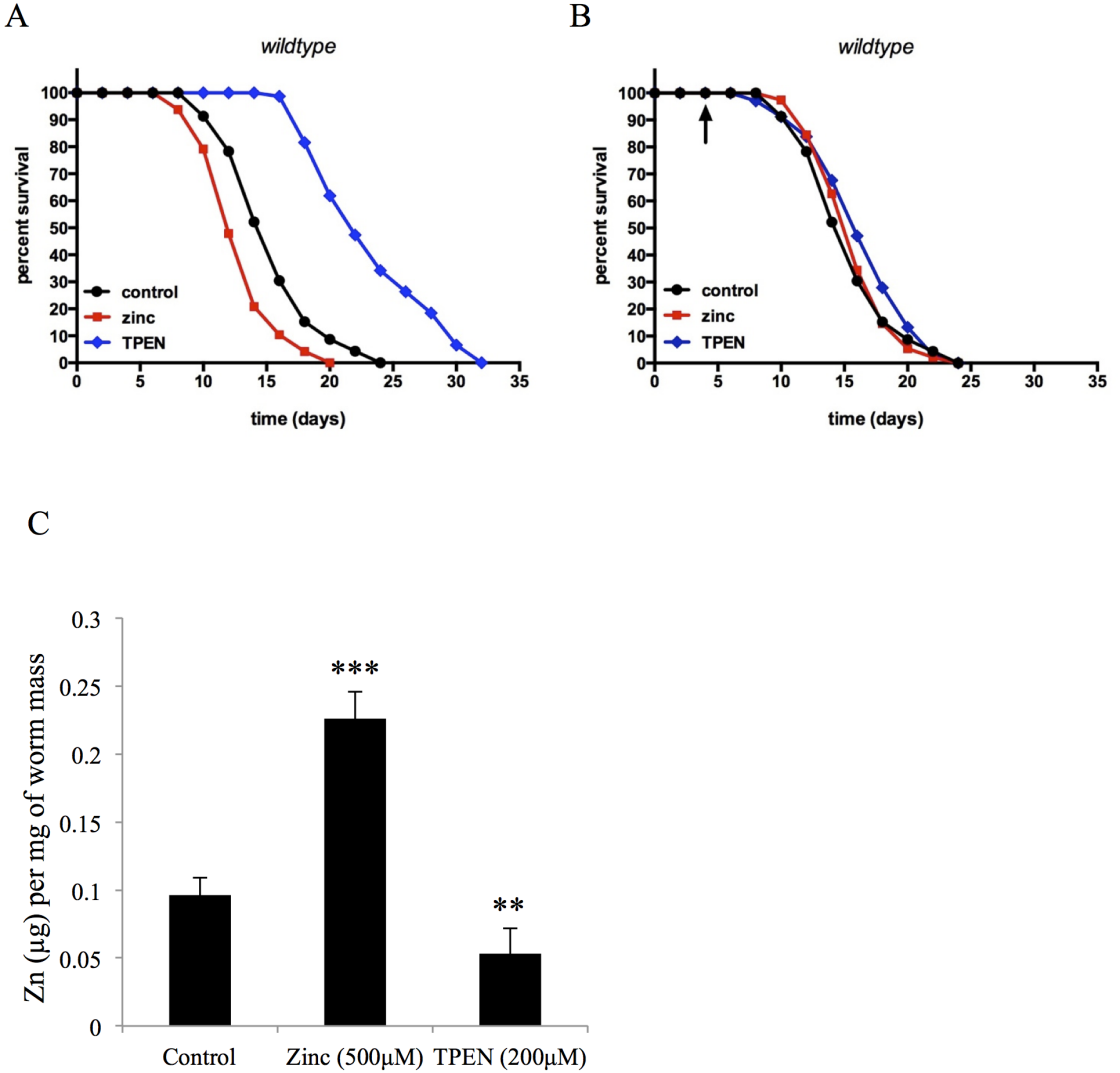
Zinc availability regulates the lifespan of *C*. *elegans*. **(A) Kaplan-Meier survival curve of worm populations exposed to zinc or TPEN as larvae**. Wildtype worms were exposed to 500μM ZnSO_4_ or 200μM TPEN at the L3 stage. Mean and maximum population lifespan were reduced with zinc and increased with TPEN treatment (p < 0.0001, log rank test) **(B) Kaplan-Meier survival curve of worm populations exposed to zinc or TPEN as adults**. Wildtype worms were exposed to 500μM ZnSO_4_ or 200μM TPEN at day 5 of adulthood. Thl.ere was no significant difference in mean and maximum population lifespan due to zinc or TPEN treatment. **(C) Changes in total zinc content in worms exposed to zinc or TPEN as larvae**. Wildtype worms were exposed to 500μM ZnSO_4_ or 200μM TPEN at the L3 stage and collected at one-day old adult animals for analysis of total zinc content. Worms supplemented with zinc had a ~2-fold increase in total zinc content (***, p<0.0001, t-test), while worms treated with TPEN demonstrated a ~2-fold decrease in total zinc content (**, p<0.001, t-test). Data shown are the mean ± SD of 3 experimental replicates.

Lifespan analysis was performed under conditions of chronic exposure to supplemental zinc. The mean life span of control wildtype worms was 16.1±0.9 days. When worms were cultured with 500μM zinc starting at the L3 development stage, the populations showed a reduced survival time of 14.3±0.4 days, representing a 14% decrease in mean lifespan (Fig 1A). The effect of zinc on population lifespan were dose dependent (S2 Fig). However, when the exposure to excess zinc was delayed until day 5 of adulthood, the worms did not show a change in lifespan (controls 14.9±0.9 days vs. zinc treated 15.4±0.7 days), suggesting that the effect of excess zinc on lifespan only occur when exposed during early development (Fig 1B). To demonstrate the lifespan effect was due to zinc and not due to the sulfate anion, testing was repeated with 500μM ZnCl_2_, which yielded comparable results to ZnSO_4_ treatment (S3 Fig).

**Fig 2:**
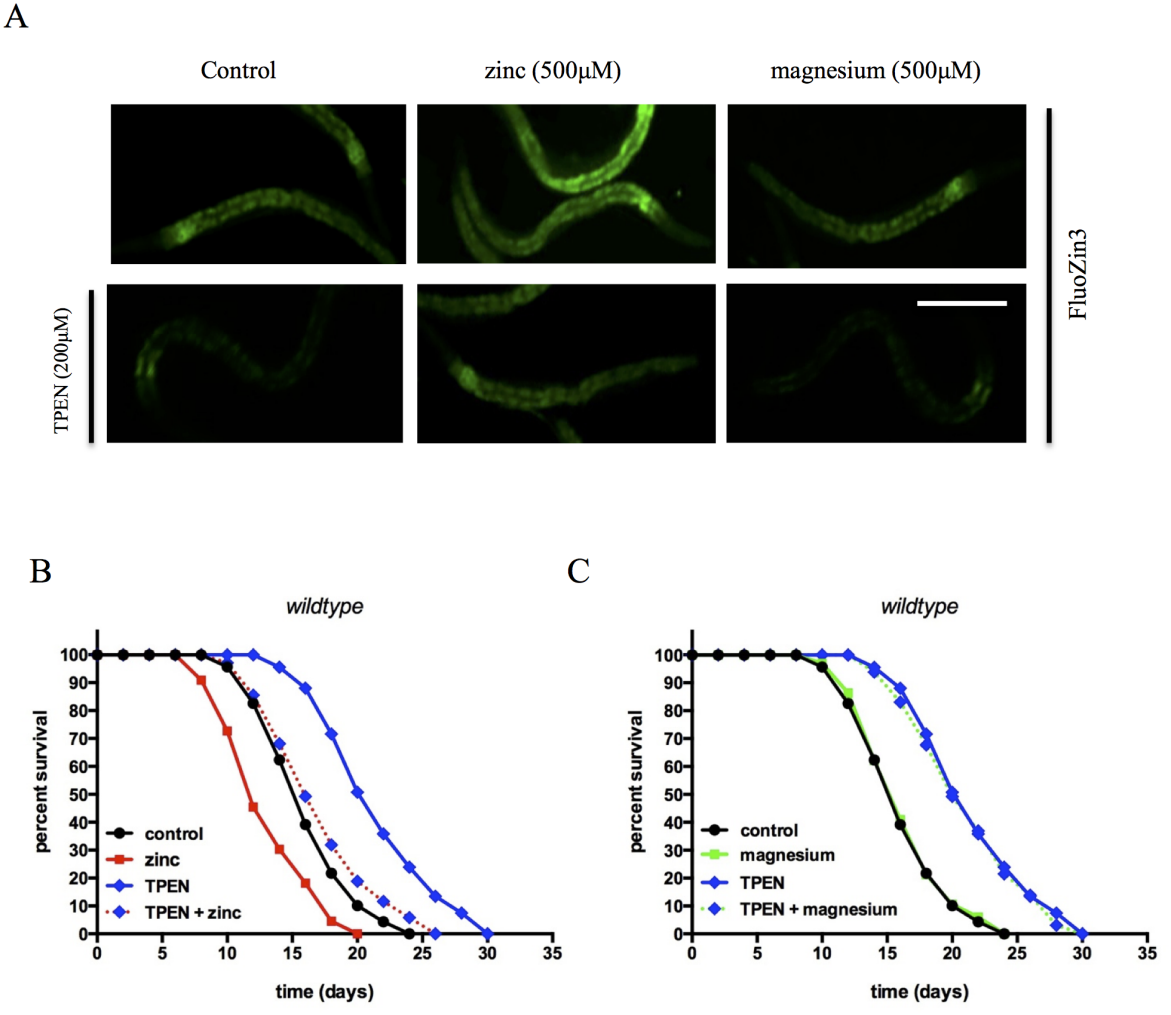
TPEN effects on *C*. *elegans* metal content and lifespan are zinc-specific. **(A) TPEN effect on labile zinc levels is specific to the chelation of zinc**. Wildtype worms were exposed to 200μM TPEN in the presence or absence of 500μM ZnSO_4_ or 500μM MgSO_4_ at L3 stage and collected at one-day old adult animals for analysis of relative labile zinc content. The representative micrograph shows that worms supplemented with zinc have elevated labile zinc content, while TPEN treatment decreased labile zinc. Equal molar levels of magnesium had no effect on basal or TPEN-induced fluorescence. Scale bar = 0.2mm. **(B) Kaplan-Meier survival curve of worm populations exposed to zinc and/or TPEN**. Wildtype worms were exposed to 200μM TPEN in the presence or absence of 500μM ZnSO_4_ at the L3 stage and monitored for effects on lifespan. Mean and maximum population lifespan were reduced with zinc supplementation and increased with TPEN treatment (p < 0.0001, log rank test). Additional 500μM ZnSO_4_ attenuated the effect of TPEN on mean and maximum lifespan. **(C) Kaplan-Meier survival curve of worm populations exposed to magnesium and/or TPEN**. Wildtype worms were exposed to 200μM TPEN in the presence or absence of 500μM MgSO_4_ at the L3 stage and monitored for effects on lifespan. Mean and maximum population lifespan were increased with TPEN treatment (p < 0.0001, log rank test). Addition of MgSO_4_ had no effect on mean and maximum lifespan.

We tested whether providing supplemental zinc through diet increased the total zinc levels within the worms using inductively-coupled plasma optical emission spectrometry (ICP-OES) [42]. Baseline zinc content was determined to be 0.09 ± 0.02 μg/mg for the L3 stage, 0.10 ± 0.02 μg/mg for 1-day old adult, and 0.14±0.03 μg/mg for 5-day old adults, so only a moderate basal increase in zinc content with age. We found that worms supplemented with 500μM zinc had a 109.0±8.9% increase in total zinc content compared to control worms (Fig 1C), but no significant change in other metal content (data not shown). To verify the specificity of the zinc effect, we used MgSO_4_ instead of zinc as an additional control, as the magnesium ion has a similar charge and ionic radius compared to zinc but does not bind to the FluoZin-3 probe [43]. MgSO_4_ supplementation did not increase FluorZin-3 fluorescence in the worms or alter the response to TPEN (Fig 2A). Additionally, it was possible that the excess zinc affected bacterial viability, causing the release of bacterial metabolites that could influence *C*. *elegans* lifespan. To test whether the zinc-dependent decrease in lifespan resulted in part from dead bacteria, lifespan assays were repeated using UV-killed OP50 *E*. *coli* for feeding. The mean lifespan of control worms was 18.6± 0.7 days compared to 16.3±0.7 days for worms cultured on supplemental zinc (12% decrease), which was similar to results from supplemental zinc with living bacteria, suggesting that altered bacterial metabolites did not explain the shortened lifespan in worms (S3 Fig).

In addition to direct supplementation of diet, we tested another method to increase zinc by feeding worms *E*. *coli* that were genetically mutated in the gene *zitB*, a zinc transporter in the bacterial cytoplasmic membrane. *ZitB* mutants cannot efficiently efflux excess zinc from within the cell, and thus have higher levels of endogenous zinc [44]. When wildtype worms were fed ZitB bacteria, mean lifespan decreased (S4 Fig), similar to worms fed wildtype bacteria plus dietary zinc supplementation. Total zinc content within the worms also increased after the worms were fed ZitB bacteria (S4 Fig), similar to control worms fed with dietary zinc supplementation. These results support a conclusion that an elevated zinc burden results in a significantly decreased lifespan in wildtype worms.

Since increasing zinc burden in worms led to a shortened lifespan, we reasoned that reducing zinc levels might have a beneficial effect. To do this, we used N, N, N′, N′-tetrakis (2-pyridylmethyl) ethylenediamine (TPEN), a membrane-permeable zinc-selective chelator that binds zinc with high affinity [32]. Previous reports have successfully used TPEN in worms up to 200μM [32], but we also tested for toxicity in our system by monitoring growth and body size development of the worms. We observed that worms cultured with TPEN up to 200μM demonstrated similar growth and body size compared to wildtype populations (S5 Fig). Compared to wildtype populations (1.07±0.12 mm), worms treated with 50μM TPEN were 1.03±0.18 mm, 100μM TPEN were 0.94±0.21 mm, and 200μM zinc were 0.85±0.28 mm long. Higher concentrations of up to 400μM TPEN showed significant reductions in growth and body size, and obvious increases in population death (data not shown).

Lifespan analysis was then performed under conditions of chronic exposure to TPEN. The mean life span of TPEN-treated worms (23.1±0.7 days) was significantly greater than control worms (16.1±0.9 days), representing a 43% increase in mean lifespan (Fig 1A). The effect of TPEN on population lifespan was dose dependent (S2 Fig). However, when exposure to TPEN was delayed until day 5 of adulthood, the worms did not show a change in lifespan, suggesting that reducing zinc has a positive effect on lifespan during early adulthood (Fig 1B). We then attempted to saturate the chelating effects of TPEN by adding additional dietary zinc (500μM). When animals were cultured with zinc (500μM) and TPEN (200μM) together, the beneficial effect of TPEN was blocked (Fig 2B). Collectively, these data show that increasing zinc levels *in vivo* by dietary supplementation decreased population lifespan, whereas decreasing zinc levels *in vivo* increased lifespan in *C*. *elegans*.

We then treated synchronous populations of worms with 200μM TPEN in NAMM media plates, seeded with OP50 bacteria for 72 hours and then harvested for ICP-OES analysis. The treated worms had a 36% decrease in zinc level (Fig 1C), but no significant change in other metal content (data not shown). We then examined the changes in zinc level *in vivo* using the FluoZin-3 zinc-selective fluorescent probe. FluoZin-3 fluorescence decreased by over 30% after treatment with TPEN (Fig 1D). Additionally, TPEN treatment effects on FluoZin-3 fluorescence were attenuated by co-treatment of zinc (Fig 2A). To verify the specificity for zinc, we substituted magnesium instead of zinc, but magnesium did not attenuate the effect of TPEN (Fig 2A and 2C).

### The effects of zinc availability on *C*. *elegans* lifespan require key transcription factors involved in longevity-determining pathways

**Fig 3:**
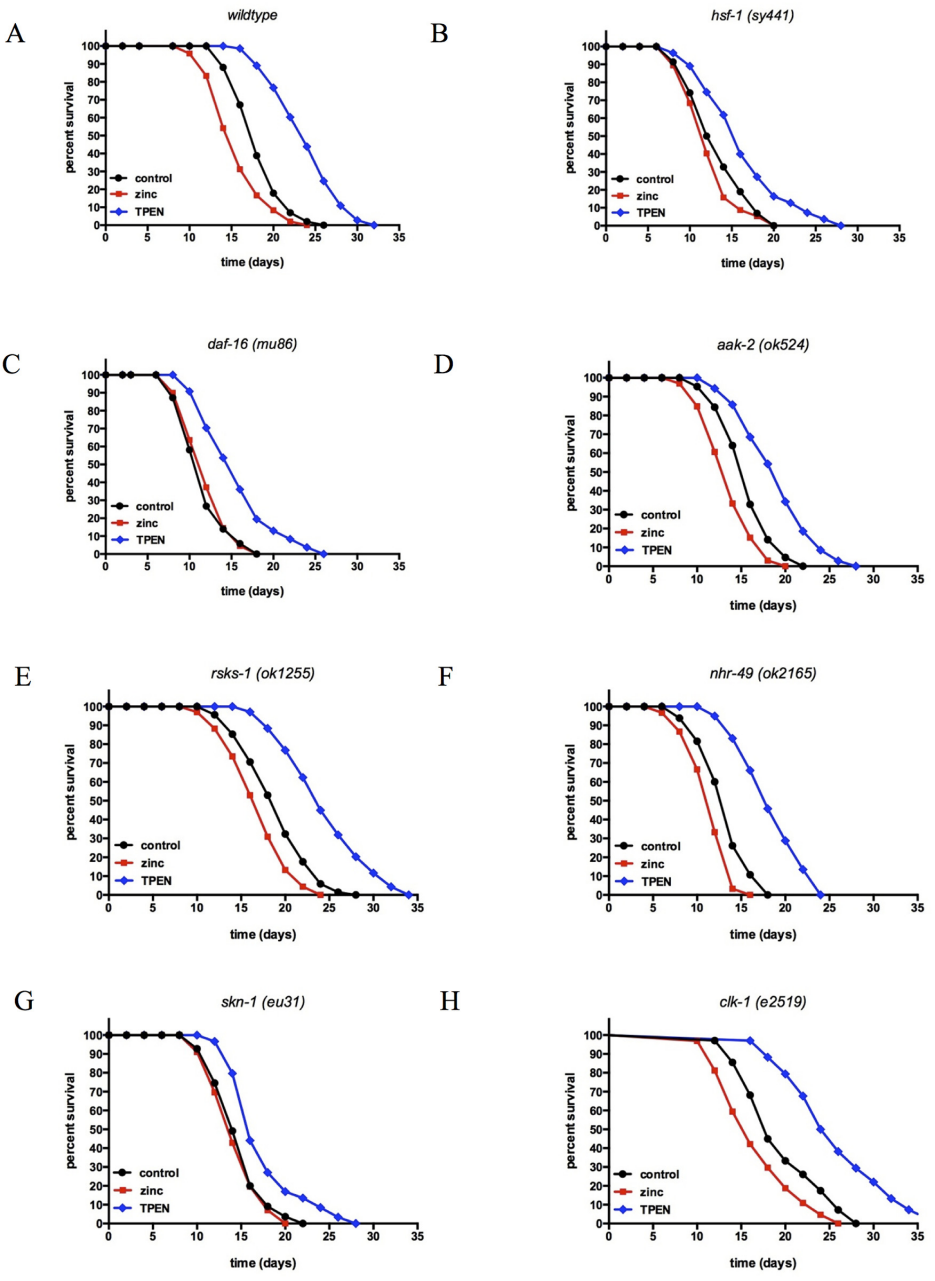
Identification of genes that modulate the effects of zinc and TPEN on lifespan in *C*. *elegans*. (A) Wildtype worms, or worms with null alleles for **(B)** *hsf-1(sy441)*, **(C)** *daf-16(mu86)*, **(D)** *aak-2(ok524)*, **(E)** *rsks-1(ok1255)*, **(F)** *nhr-49(ok2165)*, **(G)** *skn-1(eu31)*, and **(H)** *clk-1(e2519)* were exposed to 500μM ZnSO_4_ or 200μM TPEN at the L3 stage. Mean and maximum population lifespan were reduced with zinc supplementation and increased with TPEN treatment for many mutant strains, similar to wildtype worms (p < 0.0001, log rank test). However, ZnSO_4_ and TPEN-mediated changes in lifespan were attenuated in worms with mutations of *daf-16(mu86)*, *hsf-1(sy441)*, *skn-1(eu31)*.

To identify genes involved in the zinc-dependent effects on lifespan, we combined the effects of modulation of zinc levels with loss of function mutants of genes known to determine lifespan in *C*. *elegans*. We tested strains with mutations in the following genes: *daf-16*, a FOXO downstream effector molecule of insulin signaling pathway [45,46]; *hsf-1*, a heat shock factor-1 that binds heat shock response elements [47,48]; *aak-2*, an AMP-activated protein kinase that responds to the energy state of animal [49]; *rsks-1*, a putative ribosomal protein S6 kinase (S6K) involved in TOR pathway [50–53]; *nhr-49*, a nuclear hormone receptor (NHR) related to the mammalian HNF4 (hepatocyte nuclear factor 4) involved in fat metabolism [54]; *skn-1*, a putative transcription factor that promotes detoxification and stress resistance [55]; and *clk-1*, a mitochondrial protein demethoxyubiquinone (DMQ) hydroxylase necessary for ubiquinone biosynthesis [48,56]. These loss of function mutations resulted in altered baseline mean lifespans; compared to wildtype at 16.7±0.9 days, some strains had increased mean lifespan (*rsks-1* at 17% and *clk-1* at 17% increase), whereas other strains had decreased mean lifespan (*daf-16* at 28%, *hsf-1* at 20%, *nhr-49* at 20%, *aak-2* at 5% and *skn-1* at 10% decrease) (Fig 3 and S1 Table).

The effect of zinc supplementation on lifespan was then evaluated in these loss of function strains (Fig 3 and S1 Table). The addition of 500μM zinc supplementation was found to shorten the control lifespan to 14.3±0.9 (14% decrease), consistent with previous findings. Compared to the control for each loss of function strain, zinc supplementation further decreased lifespan in strains with the loss of *nhr-49* (12% decrease), *rsks-1* (12% decrease), *aak-2* (13% decrease), and *clk-1* (14% decrease). However, zinc supplementation had minimal to no effect on the lifespan in strains with the loss of *daf-16* (no change), *hsf-1* (6% decrease), and *skn-1* (2% decrease), suggesting that DAF-16, HSF-1, and SKN-1 may be required for the effects of zinc excess on lifespan. These finding also indicate that the effects of zinc excess on longevity are not simply a matter of toxicity, but are affecting lifespan through their impact on longevity signaling pathways.

The effect of zinc chelation with TPEN on lifespan was also evaluated in worms with the same loss of function strains (Fig 3 and S1 Table). The addition of 200μM TPEN was found to extend the wildtype lifespan to 22.9±1.1 (37% increase), consistent with previous findings. Compared to the control for each loss of function strain, TPEN treatment also increased lifespan in strains with loss of *nhr-49* (39% increase), *rsks-1* (27% increase), *aak-2* (23% increase), *clk-1* (33% increase), *daf-16* (30% increase), *hsf-1* (23% increase), and *skn-1* (19% increase). The lifespan extending effects of zinc chelation on were less dependent on the tested longevity genes than the lifespan shortening effects of zinc excess.

### Zinc availability regulates dauer formation in insulin/IGF-1 pathway mutant *C*. *elegans*

**Fig 4:**
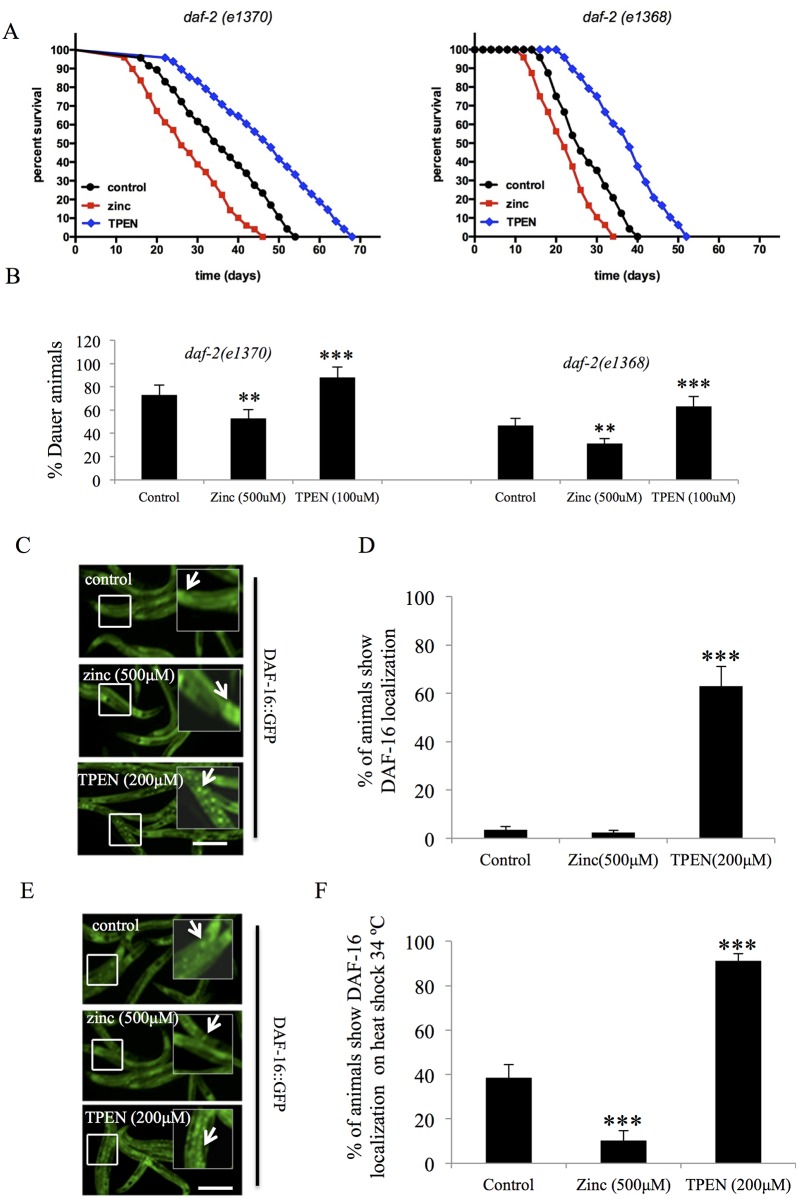
Zinc availability alters dauer formation, DAF-16 localization, and lifespan of insulin signaling pathway mutants in *C*. *elegans*. **(A) Kaplan-Meier survival curve of worm populations exposed to zinc or TPEN**. Worms with null alleles for *daf-2* were exposed to 500μM ZnSO_4_ or 200μM TPEN at L4 stage animals. Mean and maximum population lifespan were reduced with zinc and increased with TPEN treatment (p < 0.0001, log rank test). **(B) Changes in dauer formation in worm populations exposed to zinc or TPEN as larvae**. Worms with null alleles for *daf-2* were exposed to 500μM ZnSO_4_ or 200μM TPEN at L3 stage and collected at one-day old adult animals and analyzed for dauer formation. Worms supplemented with zinc had a reduced number of worms in the dauer state, whereas worms treated with TPEN had an elevated number of worms in the dauer state (**, p<0.001, ***, p<0.0001, t-test). Data indicates the mean ± SD of 3 independent experimental replicates. **(C) Imaging DAF-16 localization in worm populations exposed to zinc or TPEN at 22°C**. DAF-16::GFP transgenic worms were exposed to 500μM ZnSO_4_ or 200μM TPEN at L3 stage and measured for DAF-16 localization of one-day old adult animals. Representative micrographs showed that the subcellular localization of DAF-16::GFP was more evident in the nucleus when worms were exposed to TPEN, compared to control and zinc-supplemented worms. Scale bar = 200μm. **(D) Quantifying DAF-16 localization in worm populations exposed to zinc or TPEN at 22°C**. GFP fluorescence from DAF-16::GFP transgenice worms was imaged by fluorescence microscopy and scored for elevated nuclear DAF-16 localization. Data is represented as mean ± S.D using results of 3 experimental replicates (***, p<0.0001, log rank test). **(E) Imaging DAF-16 localization in worm populations exposed to zinc or TPEN at 34°C**. DAF-16::GFP transgenic worms were exposed to 500μM ZnSO_4_ or 200μM TPEN at L3 stage and measured for DAF-16 localization of one-day old adult animals, after exposure to heat shock at 34°C for 5 min. Representative micrographs showed that the subcellular localization of DAF-16::GFP was reduced in the zinc treated group and elevated in in the TPEN group, compared to control worms. Scale bar = 200μm. **(F) Quantifying DAF-16 localization in worm populations exposed to zinc or TPEN at 34°C**. GFP fluorescence from DAF-16::GFP transgenic worms was imaged by fluorescence microscopy and scored for elevated nuclear DAF-16 localization. Data is represented as mean ± SD using results of 3 experimental replicates (***, p<0.0001, log rank test)

In *C*. *elegans*, inhibition of insulin/IGF-1 signaling results in DAF-16 nuclear localization, resulting in dauer formation [45,46,48,57]. The dauer phenotype is a developmentally-arrested alternative larval stage of the worm that is influenced by the insulin-like peptide [57]. Having observed that altered zinc levels modulate lifespan through the insulin/IGF-1 signaling pathway, we tested the downstream effects of zinc on longevity and dauer formation using zinc supplementation or chelation in two different *daf-2* mutant worms populations. DAF-2 is an insulin/IGF receptor ortholog in *C*. *elegans*, and *daf-2* mutants demonstrate higher levels of dauer formation [48,58]. Consistent with previous reports [58], the mean lifespan of two different *daf-2* mutant alleles in control conditions was 32–35 days (110–135% increase), compared to ~15 days for control N2 strains, so we then examined the influence of altered levels of zinc. We observed that supplementation with 500μM zinc decreased lifespan (20–28%) relative whereas chelation with 100μM TPEN increased lifespan (33–58%), in *daf-2* mutant controls (Fig 4A). We also observed that supplementation with 500μM zinc decreased dauer formation (30–34%) relative to *daf-2* mutant controls, whereas zinc chelation with 100μM TPEN increased (20–32%) dauer formation (Fig 4B).

We then investigated whether the function of the DAF-16 transcription factor was altered by zinc imbalances. Upon inhibition of the insulin/IGF-1 pathway, DAF-16 translocates to the nucleus and activates specific target genes. We tested the localization of DAF-16::GFP in worms treated with zinc (500μM) or TPEN (200μM). In control and zinc-treated worms, DAF-16::GFP localization was mostly in a diffuse cytoplasmic pattern. In contrast, TPEN treatment promoted DAF-16 distinct punctate localization, suggesting nuclear translocation (Fig 4C and 4D). To further validate zinc-mediated suppression of DAF-16 nuclear entry, we heat-sensitized the DAF-16::GFP transgenic animals by exposing them to 34°C for 5 min, and examined for the localization of DAF-16::GFP in control worms and those with altered zinc levels. The percentage of worms with DAF-16 nuclear localization observed in control animals was 40±5%, compared to zinc supplemented animals 10±3%, and TPEN treated animals 95±3% (Fig 4E and 4F). The DAF-16::GFP localization studies support the idea that zinc modulates the activity of the transcription factor DAF-16 *in vivo*.

**Fig 5:**
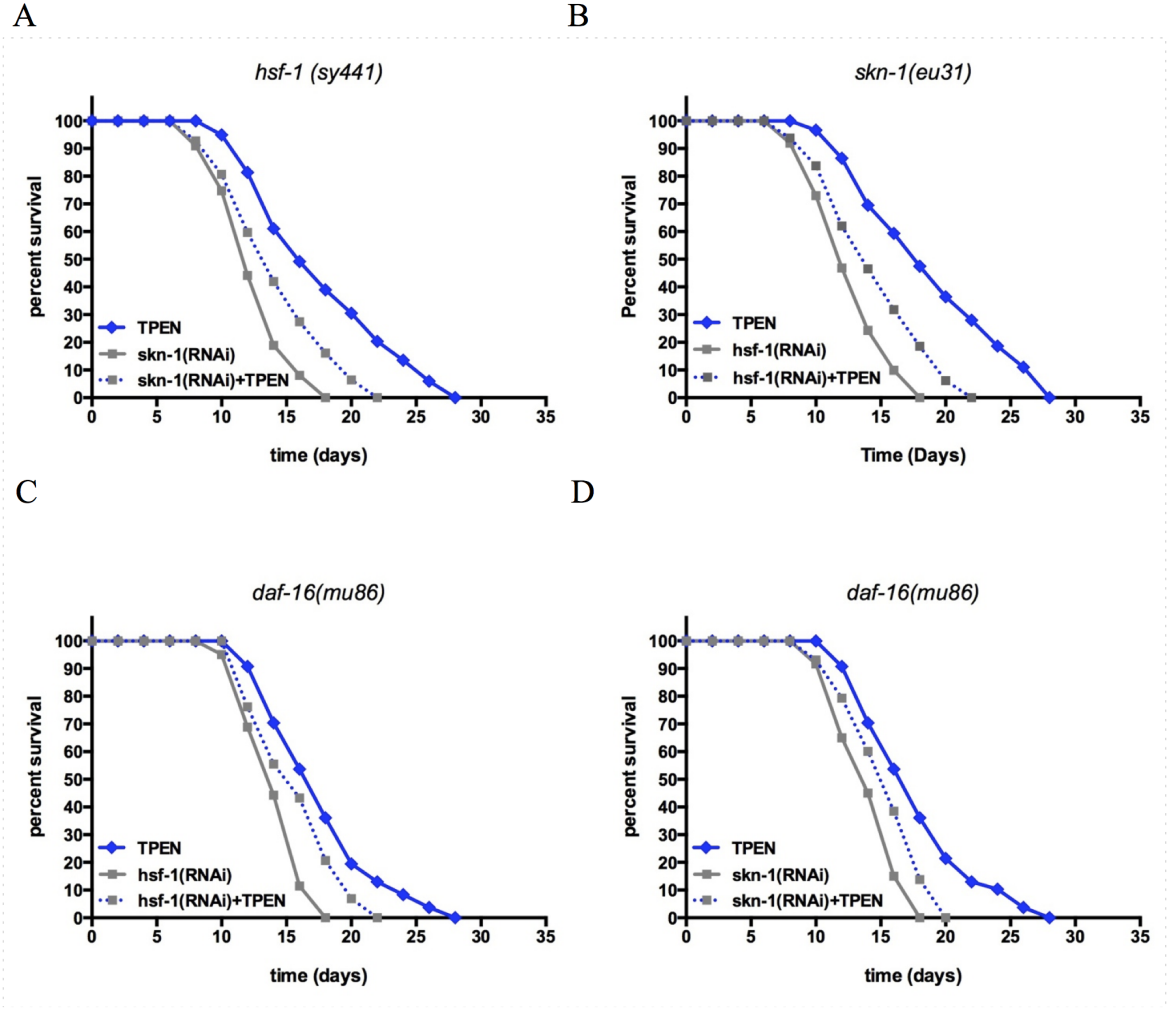
The effects of zinc availability on lifespan is dependent on HSF-1, SKN-1, and DAF-16 transcription factors in *C*. *elegans*. **(A) Kaplan-Meier survival curve of worms with knockdown of *hsf-1* exposed to TPEN as larvae**. *hsf-1* knockdown worms with or without knockdown of *skn-1* were exposed to 200μM TPEN at the L3 stage. The effect of TPEN on lifespan in this mutant was reduced when *skn-1* was also knockdown (p < 0.0001, log rank test). **(B) Kaplan-Meier survival curve of worms with knockdown of *hsf-1* exposed to TPEN as larvae**. *skn-1* knockdown worms with or without knockdown of *hsf-1* were exposed to 200μM TPEN at the L3 stage. The effect of TPEN on lifespan in this mutant was reduced when *hsf-1* was also knockdown (p < 0.0001, log rank test). **(C) Kaplan-Meier survival curve of worms with knockdown of *daf-16* exposed to TPEN as larvae**. *daf-16* knockdown worms with or without knockdown of *hsf-1* were exposed to 200μM TPEN at the L3 stage. The effect of TPEN on lifespan in this mutant was reduced when *hsf-1* was also knockdown (p < 0.0001, log rank test). **(D) Kaplan-Meier survival curve of worms with knockdown of *daf-16* exposed to TPEN as larvae**. *daf-16* knockdown worms with or without knockdown of *skn-1* were exposed to 200μM TPEN at the L3 stage. The effect of TPEN on lifespan in this mutant was reduced when *skn-1* was also knockdown (p < 0.0001, log rank test).

To further investigate whether the lifespan extended effects of TPEN, we used mutant worms or RNAi constructs such that the expression of both transcription factors, *hsf-1* and *skn-1* would be concomitantly reduced [59,60]. We examined the lifespan of double-mutant worms exposed to TPEN to reduce zinc levels *in vivo*. The double loss of function stains were significantly more effective at inhibiting the lifespan extending effects of TPEN than the single loss of function strains alone, resulting in a 15–20% reduction in mean lifespan (Fig 5A and 5B). Furthermore, we determined lifespan in worms from a *daf-16* loss of function strain on bacteria expressing RNAi construct for *hsf-1* or *skn-1* in presence or absence of 200μM TPEN. Again the double loss of function stains were significantly more effective at inhibiting the lifespan extending effects of TPEN than the single loss of function strains alone, resulting in a 9–12% reduction in mean lifespan (Fig 5C and 5D). Together these experiments suggest that the transcription factors *hsf-1*, *skn-1*, *and daf-16* are partially additive in the modulate lifespan under low zinc conditions.

### Zinc levels modulate healthspan and protein aggregation in *C*. *elegans*

Analysis of worm strains treated with zinc (up to 500μM) or TPEN (up to 200μM) did not reveal any obvious changes in gross morphology (data not shown). We then examined the locomotor behavior of wildtype worms cultured with excess zinc or TPEN as a measure of the general health of the organisms. 5, 10, and 15-day-old worms were used to analyze the number of body bends per minute to measure age-related changes in locomotion. Supplementation with 500μM zinc did not significantly affect locomotion when worms were young but decreased the locomotion behavior when worms were older (S6 Fig). Treatment with 200μM TPEN did not significantly affect locomotion when worms were young but slowed the age-related decline in locomotion behavior. This same pattern in relation to zinc burden was also observed when locomotion behavior was measured after 4 hours of heat shock stress (S6 Fig). Both locomotion behavior and thermotolerance showed responsiveness to zinc availability in *C*. *elegans*.

**Fig 6:**
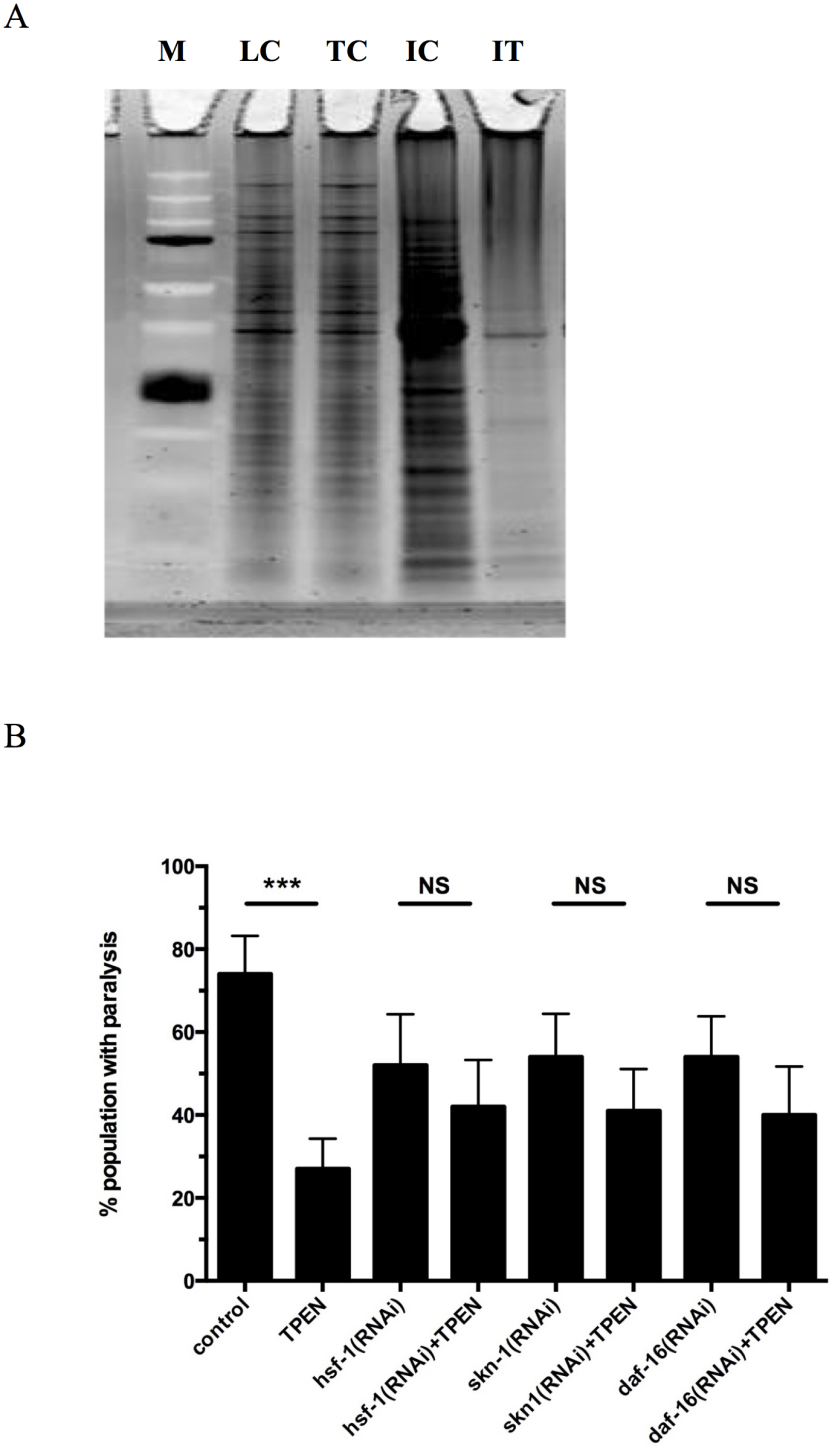
Zinc availability modulates healthspan in *C*. *elegans*. **(A) Levels of age-related protein aggregation in worm populations exposed to zinc or TPEN**. L4 stage TJ1060 [spe-9(hc88)I; fer-15(b26)II] worms were exposed to 500μM ZnSO_4_ or 200μM TPEN at L3 stage for 6 days and then analyzed for SDS-insoluble protein. The representative experiment shows worms exposed to TPEN had reduced SDS-insoluble protein in comparison with control animals. (M, marker; LC, loading control of control worms; TC, loading control of TPEN treated worms; IC, SDS-insoluble protein of control animals; IT; SDS-insoluble protein of TPEN treated worms.) **(B) Percent paralysis of worms with knockdown for specific genes supplemented with or without TPEN**. HE250 worms with knockdown for *hsf*-1(RNAi), *skn*-1(RNAi), or *daf*-16(RNAi) and with or without exposure to 200μM TPEN were scored for paralysis after 48 hours. Control worms treated with 200μM TPEN showed significantly less paralysis in comparison to control animals (***, p<0.0001, log rank test). However, TPEN was less effective when used in populations with knockdown in *hsf*-1, *skn*-1, or *daf*-16 genes (NS, not significant).

Protein homeostasis plays a major role in longevity and healthspan in many organisms. Our group and others have previously shown that insoluble protein accumulates during normal aging in *C*. *elegans* [61–66]. Since a reduction in zinc increase lifespan and healthspan in worms, we hypothesized that reducing zinc burden *in vivo* might also slow age-dependent proteostasis. We examined the amount of insoluble proteins in animals grown on control or TPEN-treated NAMM plates and found that the animals exposed to TPEN had less insoluble protein in comparison to control animals (Fig 6A). The reduction of zinc in worms reduced the amount of insoluble protein aggregates formed during normal aging.

We also determined the role of *hsf-1*, *skn-1*, *and daf-16* in relation to the effects of zinc reduction on age-dependent protein aggregation. To do this, we used the HE250 worm mutant which carries a mutation in the muscle protein UNC-52 (perlecan) that results in paralysis at 25°C [67]. Control worms treated with TPEN showed a significant reduction in paralysis compared to controls (Fig 6B and S7 Fig). However, RNAi knockdown of *hsf-1*, *skn-1*, or *daf-16* partially blocked the effects of TPEN on protein aggregation-dependent paralysis compared to untreated controls. Thus, the effects of reduced zinc that modulate proteostasis may in part depend on expression of *hsf-1*, *skn-1*, and *daf-16*.

## Discussion

Extensive studies have shown that adequate amount of different metals are required for healthy lifespan across a variety of species [68–72]. Zinc is one of the essential metals required for many biological processes in *C*. *elegans*. Under normal laboratory conditions, the dietary intake of zinc is provided by the feeding of bacteria. The worm is equipped to cope with fluctuations in zinc availability by regulating zinc uptake, sequestration, and elimination [34,35,42,73]. However, if aberrant levels of zinc persist, development and metabolism can be altered. Thus lifespan and healthspan could be affected by changes in zinc burden, but this not been fully characterized. We sought to determine the effects of zinc on *C*. *elegans* lifespan and healthspan, and how these effects are regulated by known longevity pathways.

Increasing zinc content *in vivo* using moderate levels of supplemental zinc did not result in overtly negative effects on the growth and development, yet there was a significant dose-dependent decrease in lifespan. Conversely, decreasing zinc content *in vivo* with a zinc-selective chelator resulted in significant increase in lifespan. This pattern of the detrimental effect of zinc excess and protective effect of zinc reduction was also consistent with phenotypes important in development and metabolism, including dauer formation, locomotion, thermotolerance, age-dependent protein aggregation, and age-dependent paralysis. It is unclear why *C*. *elegans* would maintain higher zinc levels under normal conditions when lower levels of zinc have longevity benefits, but there could be several reasons, including the possibility of antagonistic pleiotropy where higher zinc favors short-term benefits such as reproduction over long-term survival.

There are many known genes that play an important functional role in the regulation of lifespan in *C*. *elegans*. We tested some of these genes to determine if inactivation would block the effects of zinc excess or reduction. Our study shows that the effects of zinc imbalance on lifespan are modulated by *daf-16*, *hsf-1*, and *skn-1*. The genes *hsf-1* and *skn-1* are both part of stress defense pathways, which might be expected to be triggered when zinc levels become sub-optimum and metabolic processes become stressed. In particular, *daf-16* played a significant role in modulating healthspan by altered zinc levels. DAF-16 is a master regulator of the insulin/IGF-1 signaling pathway, which has strong control over development and aging in the worm. Zinc is known to play an important role in the formation of insulin peptide in other organisms [74]. Furthermore, zinc has been reported to regulate insulin cell signaling and mammalian target of rapamycin (mTOR) signaling [75]. Further studies require for investigate the role of zinc and its relation of mTOR pathways in *C*. *elegans*.

Two additional findings were of note in our study. First, zinc excess was shown to affect lifespan and healthspan in worms only if treated at the beginning of adulthood. Increasing or decreasing zinc levels at day 5 of life did not affect the lifespan of the worm. The reasons for this are unclear, but could involve critical development processes that render the larval worm more sensitive to the changes in zinc, or the homeostatic effectors of zinc uptake and excretion in adults are more effective than in the larval development stage. This temporal requirement of zinc on lifespan and healthspan warrants further investigation. Secondly, reducing zinc levels were shown to slow the normal age-dependent increase in protein aggregation within the worm, which is a common feature of the disease of aging. The reasons for this are also unclear, but could involve direct effects, such as the removal of excess zinc ions from aggregated proteins. In numerous other disease models, zinc has been shown to modulate protein aggregation, including the promotion of beta amyloid aggregates in models of Alzheimer disease [76,77] and even urinary concretions in a *D*. *melanogaster* [78]. Moreover, the use of zinc-selective metal chelators has been shown to be efficacious in improving proteostasis and overall morbidity [79,80]. Alternatively, the reduction in zinc could also have indirect effects, such as disruption of redox states, stimulation of oxidant defense systems, or activation of pathways that degrade protein aggregates. Our observation that inactivation of specific genes can partially attenuate the affect of zinc chelation are suggestive of this last possibility. It is likely, however, that the effects of zinc reduction are a summation of multiple pro-longevity processes, which should be defined in detail in order to develop new approaches to modulate conserved longevity determining pathways.

## Material and Method

### *C*. *elegans* strains

*C*. *elegans* strains were maintained at 20°C on nematode growth media (NGM) agar plates seeded with *E*. *coli* strain OP50 [81]. For the lifespan experiments, NAMM agar plates seeded with *E*. *coli* strain OP50 were used [36]. The following animals were used in the study: *wildtype*, *hsf-1(sy441)*, *daf-16(86)*, *aak-2(ok524)*, *rsks-1(ok1255)*, *nhr-49(ok2165)*, *skn-1(eu31)*, *clk-1(e2519)*, *daf-2(e1370)*, *daf-2(e1368)*, *muEx108 ((pKL99-2) daf-16*::*GFP/daf16bKO + rol-6(su1006))*, and HE250. All the strains were provided by the *Caenorhabditis Genetics Center* (CGC), which is funded by NIH Office of Research Infrastructure Programs (P40 OD010440). All assays utilized >/ = 300 worms per condition unless otherwise stated.

### Bacterial strain

Bacterial strains K-12 and ZitB were grown in Luria-Bertani (LB) media, and subsequently grown on minimal media (50mL 1M NaCl, 7.5mL 5M NH_4_Cl, 2mL 0.5M CaCl_2_, 25 ml 1M phosphate buffer, 10mL 40% glucose, 1mL 1 mg/mL thiamine, 1mL 1M MgSO_4_, and QS to 1L with distilled water) at 37°C. Fully grown bacteria were spotted and cultured overnight at 37°C on minimal media plates (20g agar, 50mL 1M NaCl, 7.5mL 5M NH_4_Cl, 2mL 0.5M CaCl_2_, 1mL 5 mg/mL cholesterol, 25 ml 1M phosphate buffer, 10mL 40% glucose, 1mL 1 mg/mL thiamine, and 1mL 1M MgSO_4_, and QS to 1L with distilled water). These plates were used to perform lifespan assay.

### Lifespan assay

Synchronized populations of L3 larval stage or adult worms were placed on NAMM agar plates supplemented with either ZnSO_4_ (500μM) or TPEN (200μM). The animals were transferred to fresh NGM plates every 2–3 days, and number of animals alive was scored every alternate day until death. Animals that failed to display touch-provoked movement were scored as dead. Animals that died from causes other than aging, such as sticking to the plate walls, internal hatching or bursting in the vulval region, were removed from the analysis. All lifespan experiments were performed at 20°C. Mean lifespan and number of worms used in each experiment are listed in S1 Table.

### Worm development assay

Gravid adult hermaphrodites were treated with 1N NaOH and sodium hypochlorite (1:1) to release the eggs. The eggs were transferred to NAMM agar plates seeded with OP50 and supplemented with ZnSO_4_ (200–1000μM) or TPEN (50–200μM). Plates were then incubated at 20°C. After 3 days, images of control and treated animals were counted using microscopy.

### Quantification of total zinc content

Synchronized population of L3 larval stage worms were placed on noble agar minimal media (NAMM) agar plates seeded with OP50 and supplemented with either ZnSO_4_ (500μM) or TPEN (200μM) followed by incubation at 20°C. After 48 hours, one-day adult animals were collected in M9 buffer (3g KH_2_PO_4_, 6g Na_2_HPO_4_, 5g NaCl, 1ml 1M MgSO_4_, and QS to 1L with distilled water). Worm pellets were created containing > 5000 worms each and washing 3 times with double distilled water. Tubes were incubated at 60°C to desiccate the worms and then weighted to obtain dry mass. The desiccated pellets of worms were dissolved by addition of 0.25 ml OmniTrace 70% HNO_3_ (EMD Chemicals) and incubated overnight at 60°C with 150–200 rpm orbital shaking. The acid lysates were then diluted to 5% HNO3 with OmniTrace water (EMD Chemicals), clarified by centrifugation (3000xg for 10 min), and introduced via a pneumatic concentric nebulizer using argon carrier gas into a Vista Pro inductively coupled plasma optimal emission spectrometry (ICP-OES; Varian Inc). The ICP-OES was calibrated using National Institute of Standards and Technology (NIST)-traceable elemental standards and validated using NIST-traceable 1577b bovine liver reference material. 34 elements including zinc were queried, with detection range between 0.005–50 parts per million and coefficient of variation (CV) for intra-assay and inter-assay precision typically ranging between 5%-10%. Cesium (50 ppm) was used for ionization suppression and yttrium (5 ppm) was used as an internal standard for all samples. All reagents and plasticware were certified or routinely tested for trace metal work. Elemental content data was summarized using native software (ICP Expert; Varian Inc) and normalized to dry weight of the worms.

### Labile zinc content

The L3 larval stage worms were cultured as described above with supplementation of ZnSO_4_ (500μM) or TPEN (200μM). The worms were transferred on NAMM plate and incubated for one hour to minimize the signal due to auto-fluorescence. To measure zinc *in vivo*, worms were treated with FluoZin-3 (Molecular Probes/Invitrogen), which selectively binds to labile zinc ions and allows for detection of changes in the relative labile zinc concentration inside the worm [32, 43]. For quantification of FluoZin-3, images of live animals were taken in the linear range of exposure and quantified using ImageJ (NIH) software.

### Dauer assay

Eggs were seeded on to NAMM agar plates supplemented with either ZnSO_4_ (500μM) or TPEN (100μM) seeded with OP50. Plates were incubated at 22°C for *daf-2(e1370)* and *daf-2(e1368)* worms. After 3–4 days, dauer and non-dauer worms are counted manually using a dissecting microscope. The dauer worm count was verified by treating the culture with 1% SDS for 45 min to dissolve away the non-dauer worms.

### DAF-16::GFP localization shift assay

Synchronized population of L3 larval stage worms of transgenic line DAF-16::GFP were cultured on NAMM plate supplemented with either ZnSO_4_ (500μM) or TPEN (200μM) at 25°C. One day adult animals were used to monitor cytoplasmic or nuclear localization of DAF-16. The subcellular localization of DAF-16::GFP was obtained using fluorescence microscopy with an Olympus BX51 fluorescent microscope. To sensitize DAF-16::GFP worms, the plates were shifted to 34°C for 5 min, and monitored for nuclear localization of DAF-16::GFP. Data was expressed as the mean percentage of worms that show nuclear localization of DAF-16.

### Mobility and heat stress assay

Worms were cultured on NAMM plate supplemented with either ZnSO_4_ (500μM) or TPEN (200μM) at 20°C. Treated worms were counted manually using a dissecting microscope for body bends for 10 seconds at the age of 5, 10, 15 days of adult hood and then expressed as the mean number of body bends per minute. For heat stress assay, similar age group worms were shifted to 34°C for 4 hours and then allowed to recover for 10 hours at 20°C. After recovery time, the number of live worms was counted. Three to four experimental replicates were carried out with three to four plate replicates per trial. Data was expressed as the mean number of surviving worms.

### RNAi analysis

RNA interference procedures were performed as previously described [82]. Worms were grown at 20°C and synchronized eggs were used to obtain a synchronized L3 larval stage population. L3 worms were moved to RNAi plates (NAMM containing 100mg/ml ampicillin, 20 mg/ml tetracycline, 1 mM IPTG) spotted with bacteria expressing double-stranded RNAi. The second generation animals obtained from RNAi of *hsf-1* and *skn-1* were exposed to *hsf-1*(RNAi) and *skn-1*(RNAi) with and without TPEN (200μM). The *hsf-1*(RNAi) and *skn-1*(RNAi) clones were obtained from the Ahringer library and confirmed by sequencing [83].

### Analysis of insoluble protein

Approximately 5000 synchronized population of L4 larval stage TJ1060 [spe-9(hc88)I; fer-15(b26)II][84] worms were plated on NAMM plate supplemented with TPEN (200μM) and spotted with *E*. *coli* (NA22) at 25°C (restrictive temperature). This strain does not produce fertile eggs at 25°C degree so aggregation studies will be limited to the parental generation. Worm (120-150mg total protein) were collected in M9-buffer from several plates at 6 days adulthood. Worms were washed several times with S-basal (5.85 g NaCl, 1 g K_2_HPO_4_, 6 g KH_2_PO_4_, 1 ml cholesterol (5 mg/mL in ethanol), and QS to 1 L with distilled water) and re-suspended in aqueous lysis buffer (20 mM Tris, 100 mM NaCl, 1 mM MgCl_2_, pH 7.4) with protease inhibitor cocktail (COMPLETE, Roche Diagnostics, Mannheim, Germany). The samples were sonicated (for 3 minute for 4W power, 30 cycles) on ice and then centrifuged at 3000xg to remove carcasses. All samples were normalized for total protein concentration as assessed by BCA assay (Thermo Fisher Scientific, Rockford, IL, USA) for further processing. Samples were centrifuged at 16,000xg and washed three times to extract the water-soluble protein fraction. The pellet was then re-suspended and washed three times in the same buffer containing 1% SDS to retain the detergent-soluble protein fraction. Finally, the insoluble fraction was then treated for 1 h with 70% formic acid with vigorous shaking at room temperature. The acidic fractions were concentrated in a Speed-Vac at 25°C. The samples were dissolved and SDS-PAGE was performed using 10% Bis—Tris gel NuPAGE system.

### Paralysis analysis

HE250 worm mutants which carry a mutation in the muscle protein UNC-52 (perlecan) were maintained at 16°C. Synchronized population of L3 stage HE250 worms were transferred on to NAMM media plate treated with TPEN. Plates were incubated at 16°C for 12–16 hours and then shifted to 25°C for 48 hours. After incubation for 48 hours at 25°C animals were scored for paralysis by checking for touch-provoked movement and counted manually using a dissecting microscope.

### Statistics

Survival curves were plotted and statistical analyses were performed using the Prism software (Graphpad Software, Inc., San Diego, CA, USA).

## Supporting Information

S1 Fig Effects of excess zinc on growth and development of wildtype worms. Eggs of wildtype worms were incubated at 20°C for 46 hours in the presence of ZnSO_4_ (200μM, 500μM, and 1mM) to examine the growth and development by light microscopy. The representative experiment shows the egg grown in the presence of ZnSO_4_ (200μM and 500μM) does not alter growth, while worms grown in the presence of zinc (1mM) showed retarded growth and development.
(PDF)

S2 Fig Zinc availability dose-dependently regulates the lifespan of wildtype worm populations. **(A) Excess zinc levels decreased lifespan in a concentration dependent manner**. Wildtype worms were grown in the presence of a range of ZnSO_4_ concentrations, with all tested doses significantly different from control (p<0.0001, log rank test). The apparent lowest dose that reached maximal effect was 500μM. **(B) TPEN levels increased lifespan in a concentration dependent manner**. Wildtype worms were grown in the presence of a range of TPEN concentrations, with all tested doses significantly different from control (p<0.0001, log rank test). The apparent lowest dose that reached maximal effect was 200μM.
(PDF)

S3 Fig Zinc form does not effect lifespan of wildtype worm populations. **(A) Type of zinc salt did not alter effect of excess zinc on wildtype worms**. Kaplan-Meier survival curve of wildtype worms cultured on NAMM media containing ZnCl_2_ (500μM). Treatment of zinc was initiated on L3 animals. The animals showed significant decrease in the life span on ZnCl_2_ supplement (p < 0.0001, log rank test). (B) **Role of bacterial viability on the effects of zinc on lifespan in wildtype worms**. Kaplan-Meier survival curve of wildtype worms cultured on dead bacteria, containing ZnSO_4_ (500μM). Treatment of zinc was initiated on L3 stage. The worms showed a significant decrease in lifespan (p < 0.0001, log rank test).
(PDF)

S4 Fig Zinc source dose-dependently regulates the lifespan of wildtype worm populations. (A) **Kaplan-Meier survival curve of wildtype worms cultured on K-12 and ZitB (mutant defective in zinc efflux) bacteria on minimal media plate**. The worms grown on ZitB bacteria show significantly decreased life span in comparison to wildtype K-12 bacteria (p<0.0001, log rank test). **(B) Total zinc level in worms grown on K-12 and ZitB bacteria**. Worms grown on K-12 and ZitB bacteria showed increased zinc levels in comparison to control worms grown on K-12 bacteria (p<0.0001, t-test). Data is represented as mean ± SD using results of 3 experimental replicates.
(PDF)

S5 Fig Effects of TPEN on growth and development of wildtype worms. Eggs of wild worms were incubated at 20°C for 46 hours in the presence of TPEN (50μM, 100μM, 200 μM) to examine the growth and development of the worms. The eggs grown in the presence of 50μM and 100μM TPEN does not show any significant change in growth, while worms grown in the presence of zinc (200μM) show a significant reduction in development.
(PDF)

S6 Fig Zinc availability regulates the healthspan of wildtype worm populations. **(A) Quantifying effects of zinc availability on age-related movement**. Zinc reduces, while TPEN increase, age-related decline in body bends in 5, 10, 15 days old worms. Data is represented as mean ± SD using results of 3 experimental replicates. (** = p<0.001, *** = p<0.0001, t-test) (C) **Quantifying effects of zinc availability on thermo-tolerance**. Zinc reduces, while TPEN increase, survival after 4 hours at 34°C in 10 and 15 days old worms. Data is represented as mean ± SD using results of three experimental replicates. (***, p<0.0001).
(PDF)

S7 Fig Effects of TPEN and/or knockdown of specific aging-related genes on paralysis rates in worms. HE250 worms with knockdown for *hsf*-1(RNAi) or *skn*-1(RNAi) with or without exposure to 200μM TPEN, were scored for paralysis rates in the population over a course of 120 hours.
(PDF)

S1 Table Summary of changes in mean lifespan with different genotype and zinc availability. Results of 3 independent experimental are shown. For each analysis, >300 worms were used.
(PDF)
