# Supplementary figures and images for: Zinc Levels Modulate Lifespan through Multiple Longevity Pathways in Caenorhabditis elegans
Source: PLoS One. 2016 Apr 14;11(4):e0153513. doi: 10.1371/journal.pone.0153513 (PMC4831763; doi:10.1371/journal.pone.0153513)

**Supplementary Figure 1**

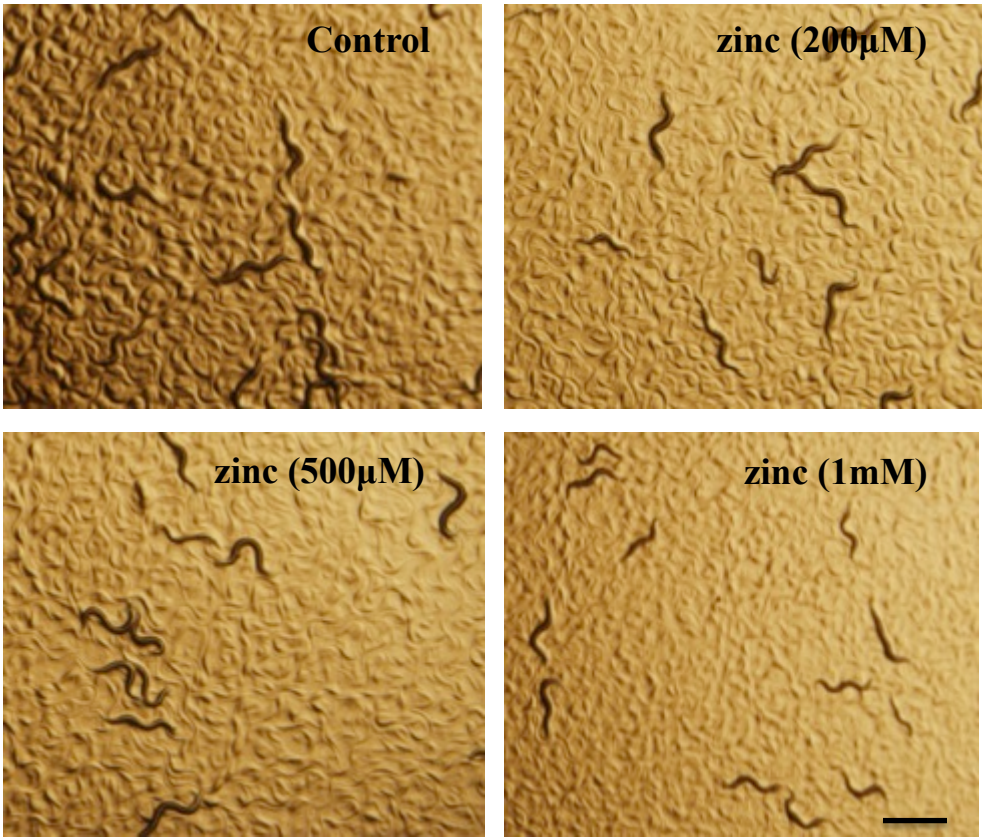

Supplement: S1 Fig — Eggs of wildtype worms were incubated at 20°C for 46 hours in the presence of ZnSO4 (200μM, 500μM, and 1mM) to examine the growth and development by light microscopy. The representative experiment shows the egg grown in the presence of ZnSO4 (200μM and 500μM) does not alter growth, while worms grown in the presence of zinc (1mM) showed retarded growth and development. (PDF) [file pone.0153513.s001.pdf]

Supplementary Figure 2

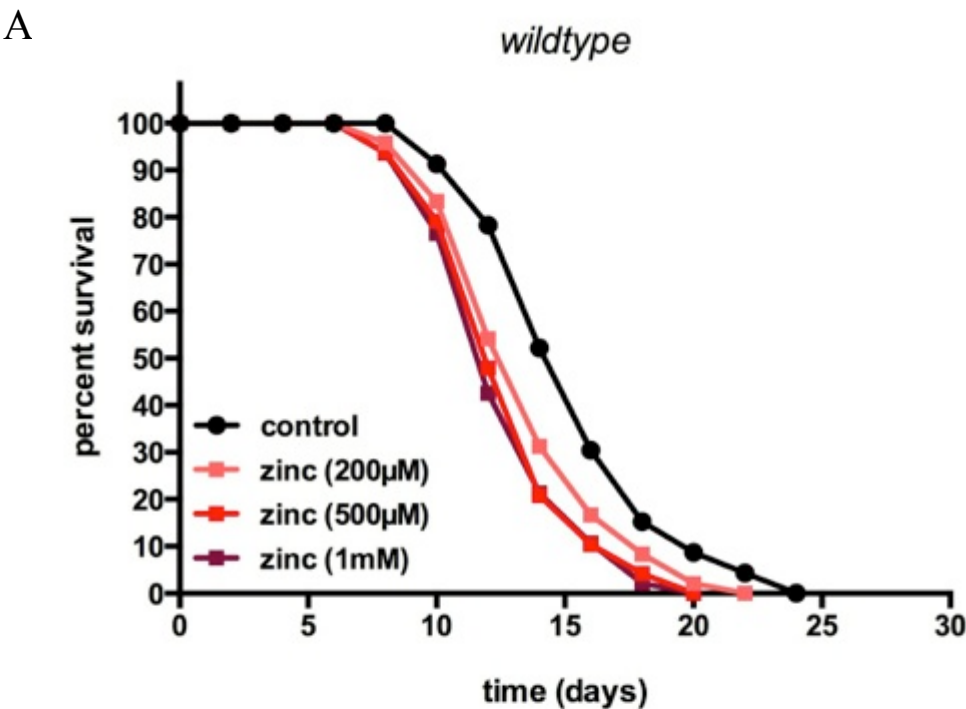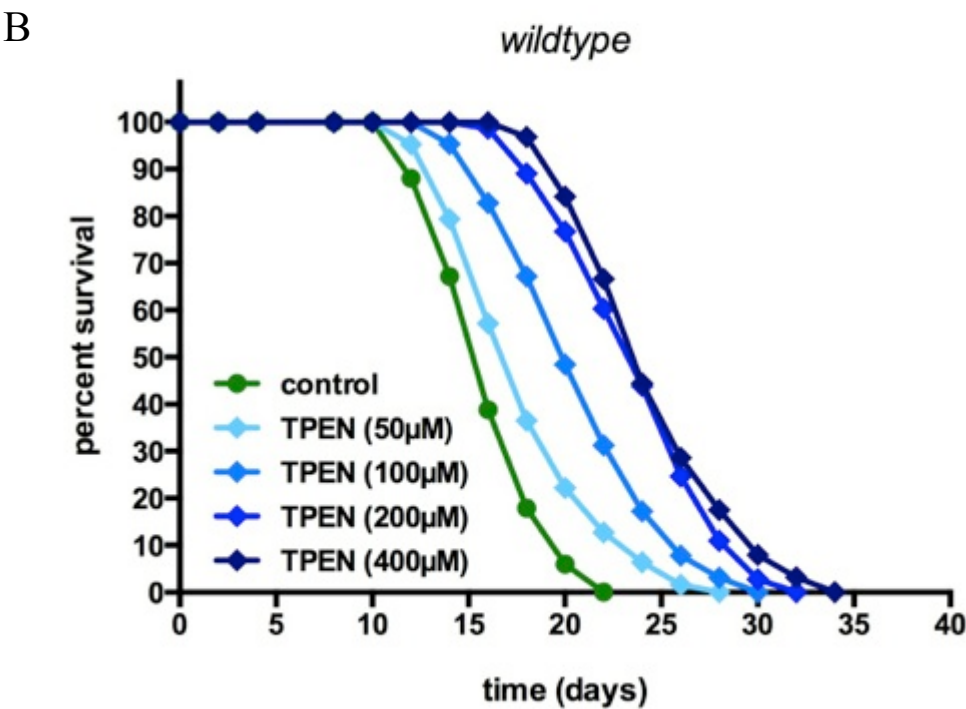

Supplement: S2 Fig — (A) Excess zinc levels decreased lifespan in a concentration dependent manner. Wildtype worms were grown in the presence of a range of ZnSO4 concentrations, with all tested doses significantly different from control (p<0.0001, log rank test). The apparent lowest dose that reached maximal effect was 500μM. (B) TPEN levels increased lifespan in a concentration dependent manner. Wildtype worms were grown in the presence of a range of TPEN concentrations, with all tested doses significantly different from control (p<0.0001, log rank test). The apparent lowest dose that reached maximal effect was 200μM. (PDF) [file pone.0153513.s002.pdf]

Supplementary Figure 3

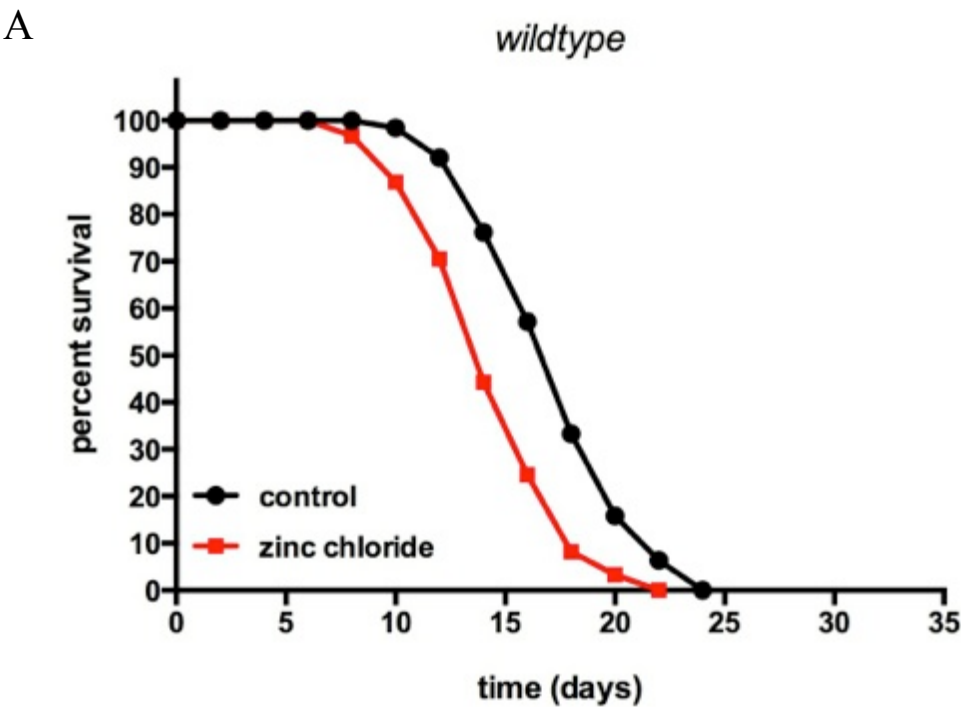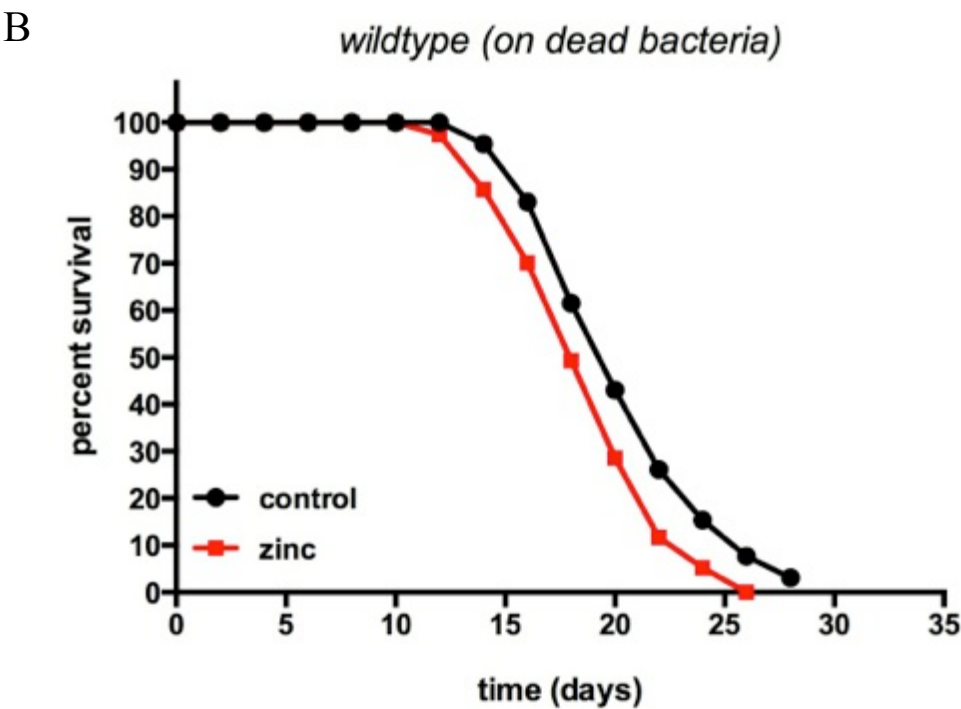

Supplement: S3 Fig — (A) Type of zinc salt did not alter effect of excess zinc on wildtype worms. Kaplan-Meier survival curve of wildtype worms cultured on NAMM media containing ZnCl2 (500μM). Treatment of zinc was initiated on L3 animals. The animals showed significant decrease in the life span on ZnCl2 supplement (p < 0.0001, log rank test). (B) Role of bacterial viability on the effects of zinc on lifespan in wildtype worms. Kaplan-Meier survival curve of wildtype worms cultured on dead bacteria, containing ZnSO4 (500μM). Treatment of zinc was initiated on L3 stage. The worms showed a significant decrease in lifespan (p < 0.0001, log rank test). (PDF) [file pone.0153513.s003.pdf]

Supplementary Figure 4

A

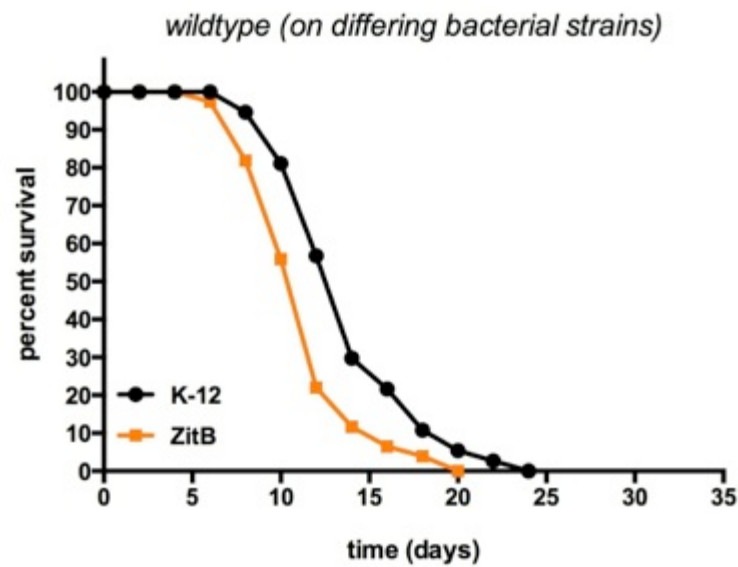

B

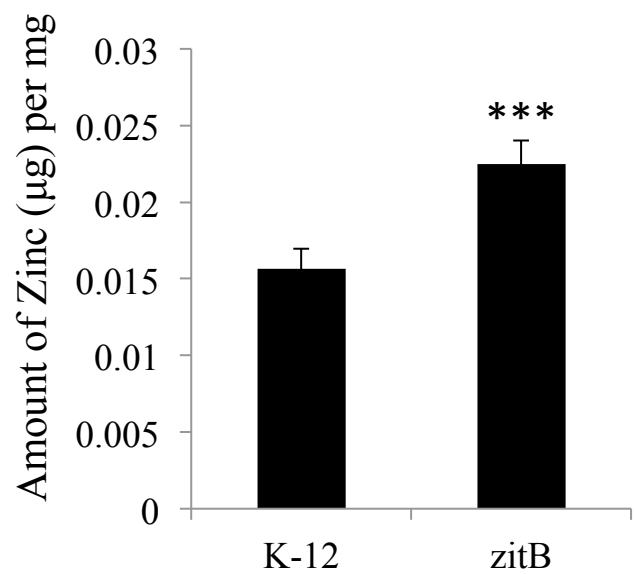

Supplement: S4 Fig — (A) Kaplan-Meier survival curve of wildtype worms cultured on K-12 and ZitB (mutant defective in zinc efflux) bacteria on minimal media plate. The worms grown on ZitB bacteria show significantly decreased life span in comparison to wildtype K-12 bacteria (p<0.0001, log rank test). (B) Total zinc level in worms grown on K-12 and ZitB bacteria. Worms grown on K-12 and ZitB bacteria showed increased zinc levels in comparison to control worms grown on K-12 bacteria (p<0.0001, t-test). Data is represented as mean ± SD using results of 3 experimental replicates. (PDF) [file pone.0153513.s004.pdf]

**Supplementary Figure 5**

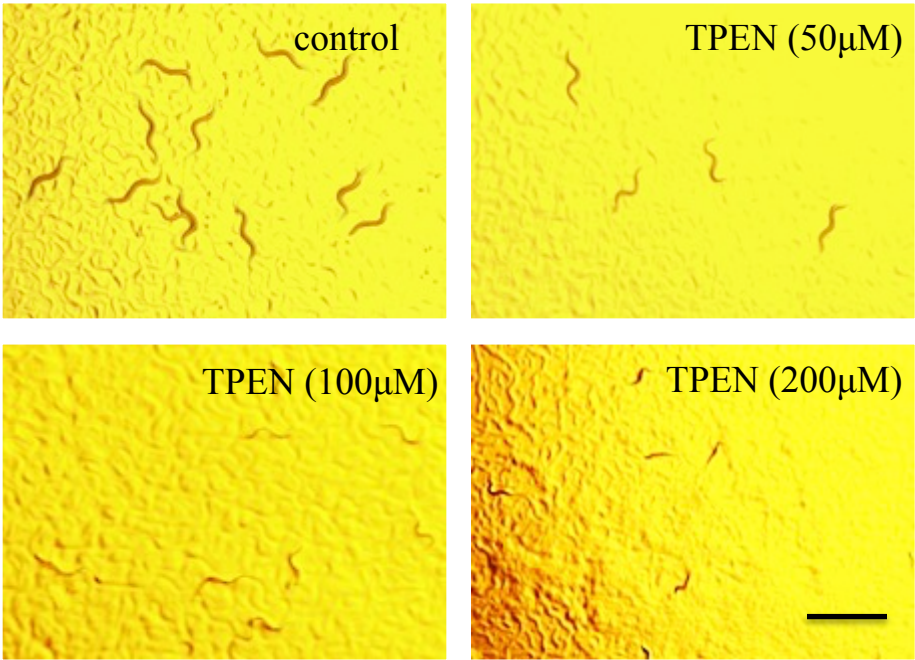

Supplement: S5 Fig — Eggs of wild worms were incubated at 20°C for 46 hours in the presence of TPEN (50μM, 100μM, 200 μM) to examine the growth and development of the worms. The eggs grown in the presence of 50μM and 100μM TPEN does not show any significant change in growth, while worms grown in the presence of zinc (200μM) show a significant reduction in development. (PDF) [file pone.0153513.s005.pdf]

Supplementary Figure 6

A

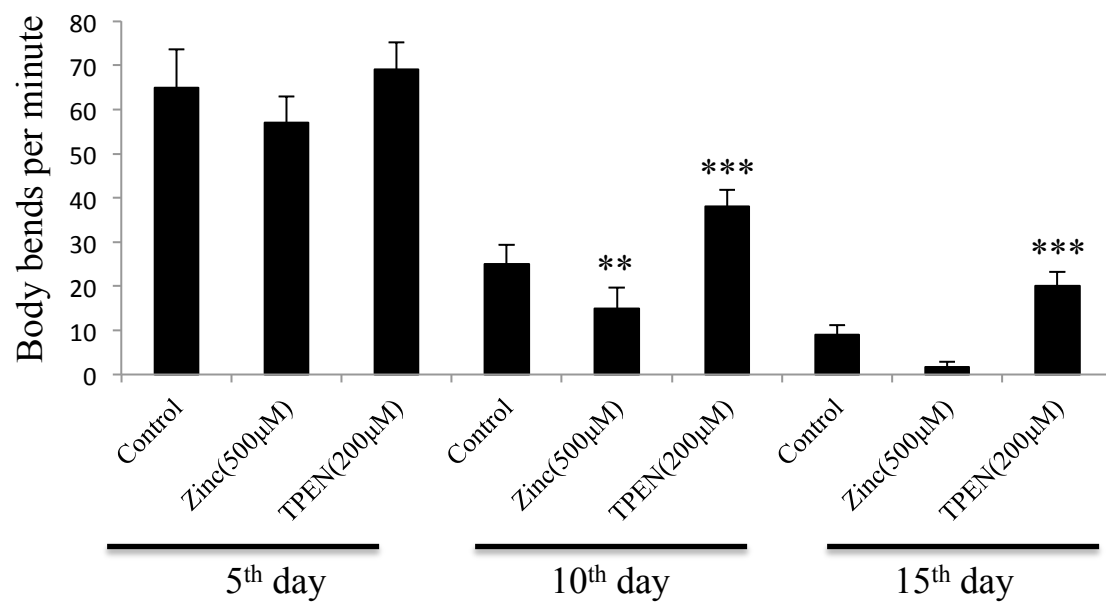

B

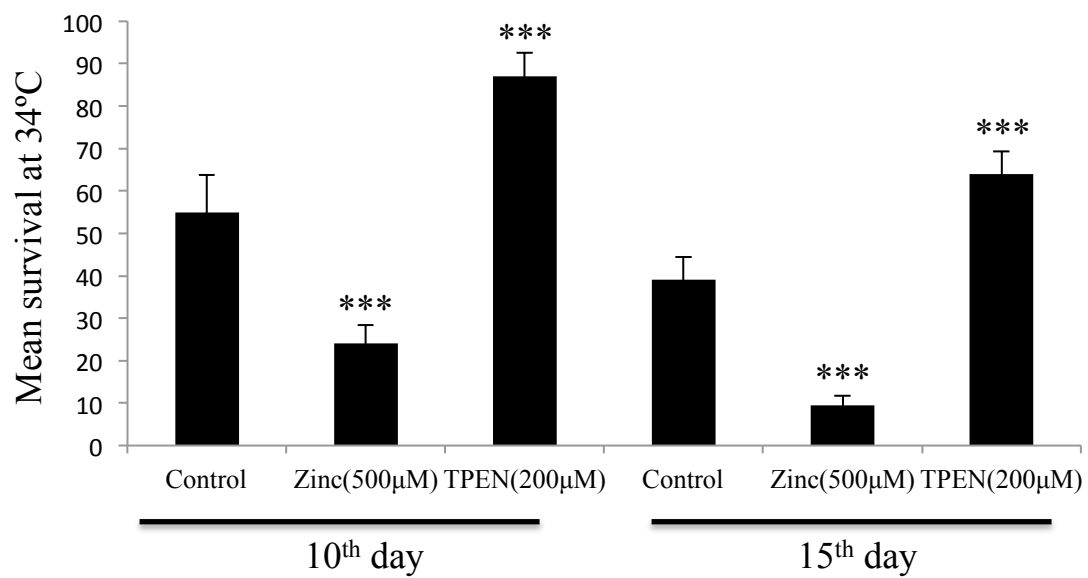

Supplement: S6 Fig — (A) Quantifying effects of zinc availability on age-related movement. Zinc reduces, while TPEN increase, age-related decline in body bends in 5, 10, 15 days old worms. Data is represented as mean ± SD using results of 3 experimental replicates. (** = p<0.001, *** = p<0.0001, t-test) (C) Quantifying effects of zinc availability on thermo-tolerance. Zinc reduces, while TPEN increase, survival after 4 hours at 34°C in 10 and 15 days old worms. Data is represented as mean ± SD using results of three experimental replicates. (***, p<0.0001). (PDF) [file pone.0153513.s006.pdf]

Supplementary Figure 7

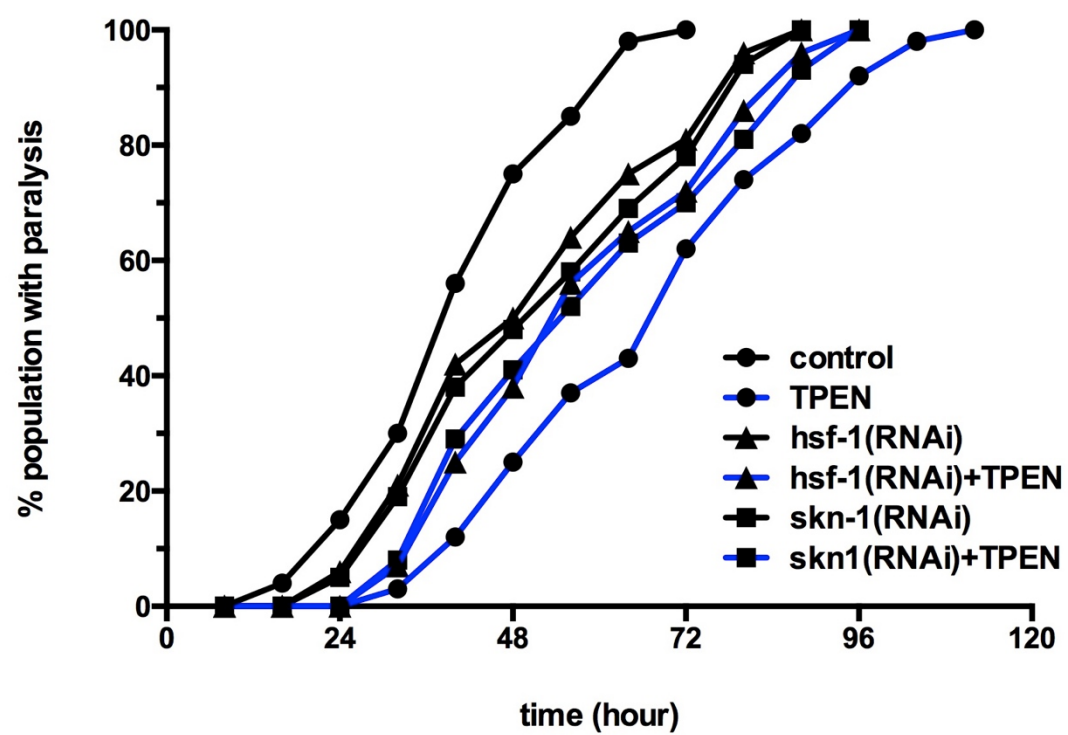

Supplement: S7 Fig — HE250 worms with knockdown for hsf-1(RNAi) or skn-1(RNAi) with or without exposure to 200μM TPEN, were scored for paralysis rates in the population over a course of 120 hours. (PDF) [file pone.0153513.s007.pdf]
