# Supplementary material for: Zinc Levels Modulate Lifespan through Multiple Longevity Pathways in Caenorhabditis elegans
Source: PLoS One. 2016 Apr 14;11(4):e0153513. doi: 10.1371/journal.pone.0153513 (PMC4831763; doi:10.1371/journal.pone.0153513)
Supplement: S1 Table — Results of 3 independent experimental are shown. For each analysis, >300 worms were used. (PDF) [file pone.0153513.s008.pdf]

Supplementary Table 1

Genotype	Condition	Mean life span ±S.D (days)	N, number of worm
Wild type	Control	16.7± 0.86	207
	Znso4 (500 µM)	14.3 ± 0.42	328
	TPEN (200 µM)	22.9 ± 1.06	249
daf-16(mu86)	Control	12.0 ± 0..33	350
	Znso4 (500 µM)	12.0 ± 0.18	277
	TPEN (200 µM)	15..6 ± 0.34	274
hsf-1(sy441)	Control	13.4 ± 0.11	226
	Znso4 (500 µM)	12.6 ± 0.45	235
	TPEN (200 µM)	16.5 ± 0.49	222
nhr-49(ok2165)	Control	13.3 ± 0.16	234
	Znso4 (500 µM)	11.7 ± 0.04	227
	TPEN (200 µM)	18.5 ± 0.16	276
rsks-1(ok1255)	Control	19.45 ± 0.30	235
	Znso4 (500 µM)	17.2 ± 0.24	261
	TPEN (200 µM)	24.7 ± 0.18	242
aak-2(ok524)	Control	15.9 ± 0.11	224
	Znso4 (500 µM)	13.8 ± 0.04	251
	TPEN (200 µM)	19.5 ± 0.85	298
skn-1 (eu31)	Control	14.9 ± 0.37	255
	Znso4 (500 µM)	14.6 ± 0.31	282
	TPEN (200 µM)	17.7 ± 0.62	243
clk-1(2519)	Control	19.5 ± 0.16	267
	Znso4 (500 µM)	16.8 ± 0.79	245
	TPEN (200 µM)	25.9 ± 0.30	229
